# Thromboinflammation and COVID-19: The Role of Exercise in the Prevention and Treatment

**DOI:** 10.3389/fcvm.2020.582824

**Published:** 2020-12-18

**Authors:** Helena Angelica Pereira Batatinha, Karsten Krüger, José Cesar Rosa Neto

**Affiliations:** ^1^Immunometabolism Research Group, Biomedical Science Institute, University of São Paulo, São Paulo, Brazil; ^2^Department of Exercise Physiology and Sports Therapy, University of Giessen, Giessen, Germany

**Keywords:** COVID-19, exercise, pandemic, thromboinflammation, cytokine storm

## Introduction

The coronavirus disease 2019 (COVID-19) pandemic is currently the biggest public health concern across the globe. On a global scale, from December 2019 to September 2020, more than 34,114,000 people were infected with the disease, with 1,016,000 deaths recorded (1). Although the etiology of the disease has long been investigated, it is still a harsh challenge for the medical and scientific community.

COVID-19 infection is complex, and the risk factors are different from the known viral respiratory infections. People with chronic inflammatory diseases (such as obesity, hypertension, diabetes, and cardiovascular disorder) are at a huge risk of developing moderate to severe symptoms and being hospitalized in the intensive care unit (ICU) (2, 3). The most common phenomena among these conditions are chronic low-grade inflammation and increased cardiovascular complications. Several evidences have been put forward to support the association between COVID-19 and thromboinflammation (3, 4). Specifically, venous thrombosis has been found to be causally related to pulmonary embolism in many cases (5).

Exercise is well-known for having a prophylactic and therapeutic effect on chronic inflammatory diseases, with a high impact on the vascular system. Furthermore, it has been reported that exercise may decrease the severity of infectious diseases and number of days of disease symptoms (6). Consistent with this, it is speculated that regular exercise represents a protective factor against the severity of COVID-19 relating to thromboinflammation and its complications.

## Exercise as a Tool for Decreasing Chronic Inflammation and Improving Angiogenesis and Immune Response

The vascular system is largely affected by COVID-19 infection. Although pulmonary failure is not directly related to the loss of pulmonary alveoli, lack of blood flow in this area can induce a collapse of the alveoli, as recently demonstrated by Ackermann et al. (7). Furthermore, kidneys are highly vascularized organs that also may be affected by this infection (2).

Venous thrombosis is usually found in coagulopathies and also observed in arterial thrombosis and stroke (7). Clinical markers of the coagulation cascade, such as D-dimer and fibrinogen, are elevated in those with moderate and severe forms of COVID-19 (8). Low innate antiviral defense and high inflammatory cytokine release contribute to the severity of COVID-19 (9), suggesting that it can be an important trigger for thrombotic complications. High amounts of pro-inflammatory cytokines contribute to the activation of thrombotic pathways. For instance, it was demonstrated that interleukin (IL)-6 induces thrombin generation and that IL-1 and tumor necrosis factor (TNF)-α inhibit anticoagulant pathways (8).

Exercise, especially in the form of regular aerobic activities, have the potential of dampening chronic inflammation by stimulating anti-inflammatory pathways and associated improvement of cardiovascular functions. Accordingly, by decreasing the basal concentration of inflammatory cytokines and reducing the percentage of pro-inflammatory T effector memory CD45+ re-expressing T cells (T-EMRA cells), exercise indirectly prevents the activation of thrombotic pathways (10).

Exercise has been shown to directly affect coagulation. While acute and strenuous activities can culminate in pro-coagulative stimuli, regular activity has been shown to diminish platelet activation under resting conditions (11). Exercise reduces fibrinogen level and enhances the plasma volume without increasing the erythrocyte volume (11). Also, exercise was used as a treatment for deep venous post-thrombotic syndrome (12). Heart failure patients with reduced fraction of ejection, when treated with moderate endurance exercise, showed a reduction in vascular endothelial damage as well as suppression of inflammation and oxidative stress (13).

The intensity and duration of aerobic exercise are correlated with the increase in nitric oxide production and reduction of reactive oxygen species, which lead to an improvement in endothelial function. Moreover, aerobic exercise reduces hypertension on coronary arteries and vascular stiffness (14).

**Figure 1:**
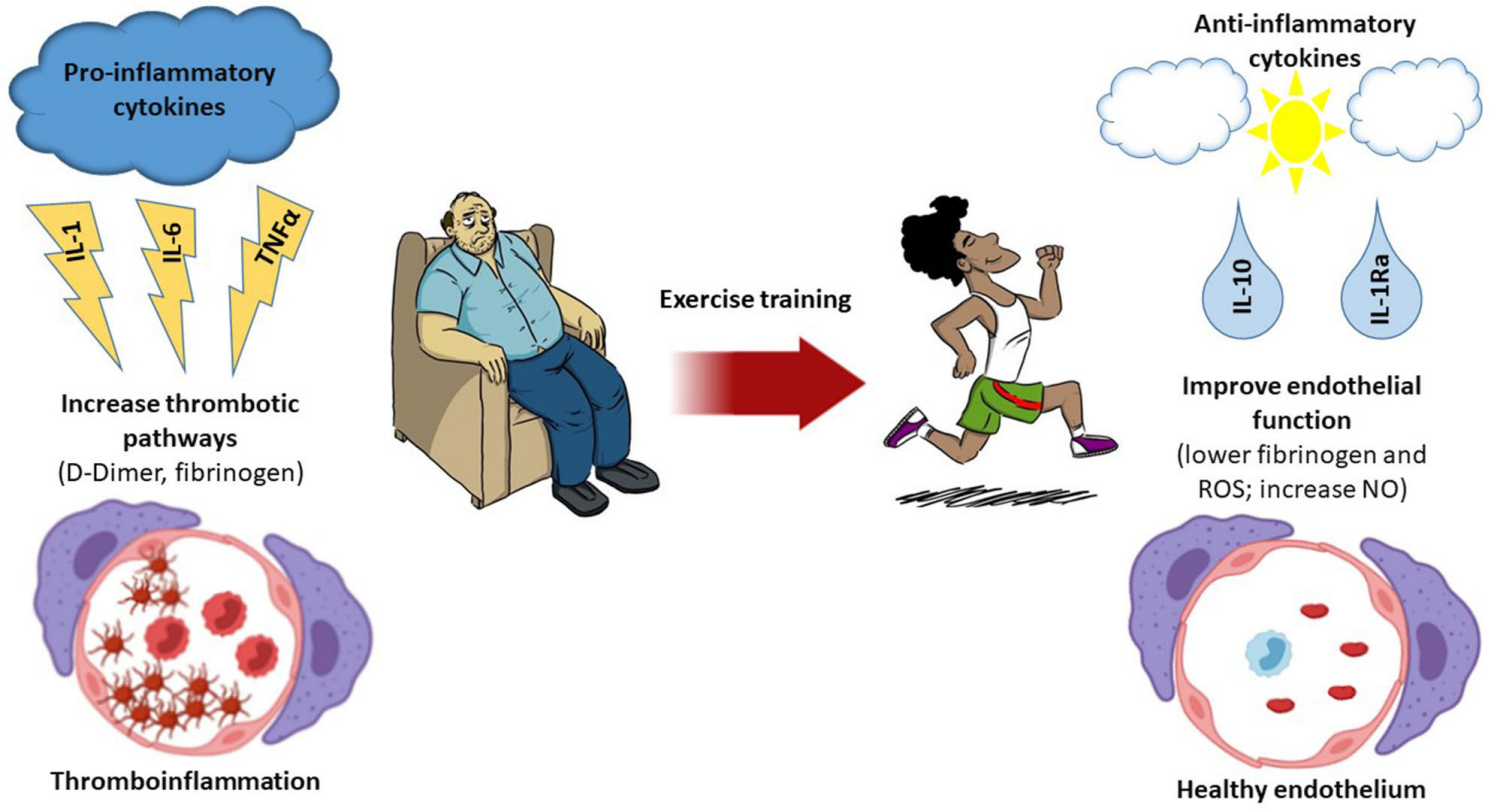
Thromboinflammation and the effect of exercise. A sedentary lifestyle leads to an increase in the release of pro-inflammatory cytokines, which induce a low-grade chronic inflammation. These inflammatory mediators enhance the thrombotic pathways that facilitate thromboinflammation, which has been associated with poor prognosis in coronavirus disease 2019 (COVID-19) patients. Exercise decreases inflammation by many pathways, including the release of anti-inflammatory cytokines. Regular exercise is associated with lower levels of fibrinogen and reactive oxygen species and increased amounts of nitric oxide (NO) production, thus inducing a healthy endothelium environment.

In parallel, regular exercise can enhance the innate and adaptive immune defense system, thus improving the response against viral infections. While it can only be speculated that exercise has a protective effect against severe acute respiratory syndrome coronavirus 2 (SARS-CoV-2) infection, regular activity has been shown to decrease the severity of infectious episodes and number of days of the symptom in other infectious diseases (6). Concerning influenza infection, exercise is associated with a lower excess risk of mortality (15). Similarly, in murine models, it was proven that moderate exercise reduces mortality in the initial days after an influenza virus infection (16). Moreover, moderate aerobic training has been shown to enhance T cell count, which is found to be decreased in the blood of SARS-CoV-2-infected patients (21), increase anti-inflammatory cytokines, improve endothelial function, and repair (Figure 1), enhance VO2peak, and have beneficial effects on clinical outcomes (22). A minimum of 150 min per week (30 min−5 days/week) of moderate aerobic exercise (5–7 on a scale of 0–10, where 0 is super easy and 10 is exhaustive) was recommended by the American College of Sports Medicine to achieve the health benefits of exercise. Moderate aerobic exercise is applied to improve immunity and metabolic complications that can reduce the poor prognosis of COVID-19 (23).

Therefore, we hypothesized that moderate intensity of aerobic training could be a protective factor against severe courses of COVID-19 (17) (Figure 1). Therefore, we can draw the attention of physicians toward assessment of the fitness level of COVID-19 patients.

## The Potential Role of Exercise in the Recovery of Those Infected With Coronavirus Disease 2019

In 2016, the WHO proposed “functioning” as a third clinical outcome indicator, such that diseases that are not fully cured are accompanied by some dysfunctions. Improving functional life while recovering from a disease is a key sign of medical effectiveness and overall health. Many patients who are recovering from COVID-19, especially those presenting severe symptoms during the infection phase, are not able to return to the normal life of caring for themselves after being discharged (18).

As discussed above, poor vascularization could cause alveoli collapse, thus leading to pulmonary failure. Several individuals infected by SARS-CoV-2 have presented respiratory problems with impairment of pulmonary ventilation function and air exchange in the alveoli, which lead to chest tightness, dyspnea, and pulmonary fibrosis (18). Pulmonary fibrosis is directly associated with high mortality rates. Furthermore, dyspnea, which is often associated with loss of skeletal muscle mass, is responsible for a decreased exercise capacity due to a reduction of daily leaving activities (19).

Several studies have investigated the role of exercise in the treatment of chronic lung disease and pulmonary fibrosis patients. A meta-analysis recently published stated that aerobic training significantly improves exercise capacity and health-related quality of life of patients with chronic respiratory disease and/or pulmonary fibrosis and that aerobic training improved the dyspnea scores when combined with breathing exercises (20).

It is important to remember that most of the benefits promoted by physical exercise in the rehabilitation of respiratory and cardiovascular diseases can be gradually lost if the patient does not continue to exercise in the long run (18). However, the practice of exercise for the improvement of medical conditions should be supervised. In conclusion, regular exercise could be an adjuvant for the prevention and treatment of COVID-19.

## Author Contributions

All authors listed have made a substantial, direct and intellectual contribution to the work, and approved it for publication.

## Conflict of Interest

The authors declare that the research was conducted in the absence of any commercial or financial relationships that could be construed as a potential conflict of interest.
